# APOE genotype modifies benefits of calorie restriction against aging

**DOI:** 10.1002/alz.085702

**Published:** 2025-01-03

**Authors:** Wenhui Qiao, Yixing Chen, Yingxue Ren, Tadafumi C Ikezu, Stephen Johnson, Fuyao Li, Kai Chen, Meixia Pan, Sonu Kashyap, Katrina Rae Bueser, Allison Wetmore, Aishe I. Kurti, Axel D. Meneses, Jada A. Owens, Eduardo N. Chini, Jun Chen, Xianlin Han, Guojun Bu, Na Zhao

**Affiliations:** ^1^ Mayo Clinic, Jacksonville, FL USA; ^2^ University of Texas Health Science Center at San Antonio, San Antonio, TX USA; ^3^ University of Texas Health Sciences Center, San Antonio, San Antonio, TX USA; ^4^ The Hong Kong University of Science and Technology, Hongkong, Hongkong China

## Abstract

**Background:**

The efficacy of Calorie Restriction (CR) in enhancing cognition, promoting healthy aging, and extending lifespan is well‐established. Yet, it remains unclear whether the apolipoprotein E (*APOE*) genotype, a known modifier for aging and age‐related disorders, influences the beneficial effects of CR in countering aging.

**Methods:**

To investigate this question, we utilized humanized APOE mouse models, which express *APOE2*, *APOE3*, or *APOE4* alleles systematically (refer to as E2, E3, and E4 mice). These mice were subjected to either ad libitum (AL) feeding or 30% CR feeding, starting from 12 months and continuing up to 20 months of age (N = 11‐20/ genotype /group, mixed sex). We monitored energy expenditure using the Comprehensive Laboratory Animal Monitoring System (CLAMS). Additionally, we evaluated anxiety levels and memory capabilities through various behavioral tests. Finally, we collected brain, blood, and feces samples for transcriptomic, lipidomic, and microbiomic analyses.

**Results:**

CLAMS measurements showed that CR significantly decreased the metabolic rate in E3 and E4 mice, but not E2 mice, during the non‐feeding period of the CR group, compared to the AL group. Furthermore, CR reduced anxiety and enhanced associative memory in E3 and E4 mice relative to the AL group. Brain transcriptomics revealed that CR upregulated the cholesterol synthesis pathway, potentially improving myelination‐related pathways in E3 and E4 mice. This finding was further validated by the increased staining intensity of myelin basic protein (MBP) observed in E3 and E4 mice from an independent cohort. In contrast, these effects were not pronounced in E2 mice. Additionally, while CR’s impact on the brain lipid profile was minor across all *APOE* genotypes, it significantly altered blood lipid profile of E2 mice, with minimal changes observed in E3 and E4 mice. Finally, in microbiomics, CR led to notable changes in the abundance of Bifidobacterium species in E3 and E4 mice and Lactobacillus species in E2 mice.

**Conclusions:**

Our study highlights the role of *APOE* genotypes in modulating the diverse effects of CR on cognition, brain transcriptomics, lipid metabolism, and the microbiome, providing a crucial foundation for considering *APOE* genotypes in developing prevention strategies for aging and age‐related disorders.

